# *Cliona varians*-Derived Actinomycetes as Bioresources of  Photoprotection-Related Bioactive End-Products

**DOI:** 10.3390/md19120674

**Published:** 2021-11-27

**Authors:** Jeysson Sánchez-Suárez, Luisa Villamil, Ericsson Coy-Barrera, Luis Díaz

**Affiliations:** 1 Doctorate in Biosciences, School of Engineering, Universidad de La Sabana, Chía 250001, Colombia; jeyssonsasu@unisabana.edu.co; 2 Bioprospecting Research Group, School of Engineering, Universidad de La Sabana, Chía 250001, Colombia; luisa.villamil@unisabana.edu.co; 3 Bioorganic Chemistry Laboratory, Universidad Militar Nueva Granada, Cajicá 250247, Colombia; ericsson.coy@unimilitar.edu.co

**Keywords:** photoprotection, sunscreen, sponge, actinobacteria, *Gordonia*, *Micrococcus*, *Promicromonospora*, *Streptomyces*

## Abstract

Sunscreen and sunblock are crucial skincare products to prevent photoaging and photocarcinogenesis through the addition of chemical filters to absorb or block ultraviolet (UV) radiation. However, several sunscreen and sunblock ingredients, mostly UV filters, have been associated with human and environmental safety concerns. Therefore, the exploration and discovery of promising novel sources of efficient and safer compounds with photoprotection-related activities are currently required. Marine invertebrates, particularly their associated microbiota, are promising providers of specialized metabolites with valuable biotechnological applications. Nevertheless, despite Actinobacteria members being a well-known source of bioactive metabolites, their photoprotective potential has been poorly explored so far. Hence, a set of methanolic extracts obtained from *Cliona varians*-derived actinomycetes was screened regarding their antioxidant and UV-absorbing capacities (i.e., photoprotection-related activities). The active extract-producing strains were identified and classified within genera *Streptomyces, Micrococcus, Gordonia,* and *Promicromonospora.* This is the first report of the isolation of these microorganisms from *C. varians* (an ecologically important Caribbean coral reef-boring sponge). The in vitro cytotoxicity on dermal fibroblasts of oxybenzone and the selected active extracts revealed that oxybenzone exerted a cytotoxic effect, whereas no cytotoxic effect of test extracts was observed. Accordingly, the most active (SPFi > 5, radical scavenging > 50%) and nontoxic (cell viability > 75%) extracts were obtained from *Streptomyces* strains. Finally, LC-MS-based characterization suggested a broad chemical space within the test strains and agreed with the reported streptomycetes’ chemodiversity. The respective metabolite profiling exposed a strain-specific metabolite occurrence, leading to the recognition of potential hits. These findings suggest that marine *Streptomyces* produce photoprotectants ought to be further explored in skincare applications.

## 1. Introduction

Chronic unprotected exposure to the sun is associated with the development of skin disorders such as altered immunity, photoaging, and cancer [1,2]. In the latter case, epidemiological surveillance has shown alarming trends [3,4] that claim more attention to photoprotective behavior. One of the primary photoprotection strategies is the application of sunscreen and/or sunblock [5]. However, several sunscreening and sunblocking agents currently employed in photoprotective product manufacture have relevant safety concerns [6,7] and even adversely affect ecosystems [8,9].

The main goal of sun protectants is oriented to avoid the harmful effects of ultraviolet (UV) radiation (i.e., photodamage-related issues). Such a goal is commonly achieved by using chemical filters, whose purpose comprises specific actions based on absorbing, reflecting, and/or scattering UV radiation [10]. However, since photodamage can be generated by different mechanisms [11], other agents such as antioxidants have recently been added to improve the photoprotection efficacy of photoprotective products [12]. This requirement for mitigating UV-induced skin damage might explain the growing demand for topically applied biologically active ingredients [13,14]. Considering the limitations regarding UV filters toxicity (for both human and environmental health) and their partial photoprotection capability, there is a reasonable demand to find new photoprotectants.

During the search for new chemical entities, natural resources continue to offer a promising opportunity to discover biologically active compounds that satisfy humanity’s demands [15]. Among the varied environment options to be explored, the marine biosphere comprises an attractive choice due to the biodiversity housed in these habitats [16]. Marine sessile invertebrates have proved to be a priceless supply of compounds with a comprehensive chemodiversity and, consequently, a broad bioactivity profile [15]. In this sense, sponges are recognized as reliable reservoirs since respective chemical and biological campaigns led to the isolation and characterization of several compounds that have been the direct or indirect basis for the development of important drugs [17].

Marine sessile invertebrates, particularly sponges, are undeniable sources of bioactive compounds [18]. In several cases, the biosynthesis of these bioactive compounds has been demonstrated to be associated with their symbiotic microorganisms [18]. *Cliona varians* is a boring encrusting sponge playing crucial ecological niches in coral reefs [19]. Bioprospecting studies on *C. varians* are scarce, and most of them have focused on their ecological roles. Recently, the bacterial diversity of *C. varians* was reported [19], and a high microbial abundance was revealed [20], including actinomycetes [19]. Despite the microbial and chemical richness exhibited by marine sponges, the development of commercially viable products (e.g., drugs, cosmetics) is limited by varied factors, namely complex structures that hinder their chemical synthesis, the low available amount of the targeted compound and the “supply problem” [21,22,23]. These challenges have led to distinct approaches to exploit the respective chemical space available from these invertebrates, such as the isolation and fermentation of their associated microbiota for bio and chemoprospecting initiatives [24,25]. In fact, this microbiota plays a relevant role in the metabolite regulation and chemodiversity described for the respective hosts (e.g., sponges) [26]. In several cases, the symbiotic microbes are the genuine source of the metabolite initially isolated from the macroorganism [24]. Consequently, bioprospecting research focused on microorganisms is currently attracting significant attention [27].

Regarding microorganisms, members of the phylum Actinobacteria are well-known for their contribution to the natural products field [28]. They are also recognized for establishing symbiotic relationships [29], including sponge-associated microbiota that is known to involve producers of active compounds of biotechnological interest [30]. Actinobacteria represent an intriguing taxon that encourages more research initiatives exploring their specialized metabolism. In this regard, the genus *Streptomyces* (class Actinobacteria) is positioned within the most prolific microbes providing drugs, mostly antibiotic agents [28]. Even though *Streptomyces’* specialized metabolism has been intensely investigated, several niches remain unexamined, particularly the symbiotic ones [29,31]. Indeed, since *Streptomyces* have traditionally been those actinobacteria that have the largest record on a substantial bioactive compounds’ repertoire, we have previously reviewed their potential as a source of photoprotective substances [32].

Therefore, as part of our interest in bioactive compounds from actinomycetes associated with marine holobionts, we aimed to identify actinobacteria strains isolated from the marine sponge *Cliona varians,* producing metabolites with photoprotection-related activities. The results revealed that different genera of the Actinobacteria class are part of the associated microbiota of *C. varians*. Additionally, the strains exhibited strain-specific metabolite occurrence. In this regard, streptomycetes showed great potential in producing bioactive compounds, specifically photoprotection-related activities. This study contributes to discovering and recognizing new bioresources to explore the presence of metabolites with an interesting bioactive profile applicable in the future to the skincare and cosmetics fields.

## 2. Results

**Figure 1:**
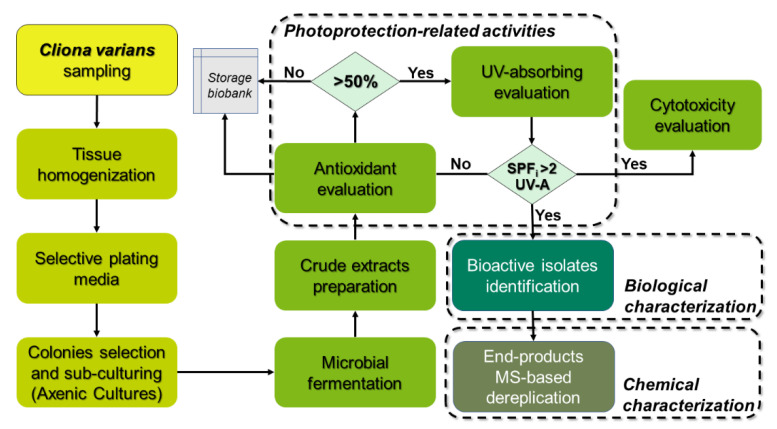
Workflow diagram showing from the biological sample collection to its characterization.

To investigate the potential of *C. varians*-associated actinomycetes as producers of metabolites with photoprotection-related activities (i.e., antioxidant and UV-absorbing capacities), we evaluated methanolic extracts (i.e., end-products) from 35 morphologically distinct isolates. The cut-off score for the antioxidant capacity (assessed by DPPH^•^ and ABTS^•+^ radical scavenging assays) was fixed at 50%, whereas for the UV absorbing, we selected those end-products with the in vitro Sun Protection Factor (SPFi) > 2 and UV-A protection capabilities (i.e., critical wavelength higher than 370 nm and UVA/UVB ratio ≥ 0.7 [33]). As a result, ten isolates were selected for biological characterization through 16S rRNA gene sequencing and chemical characterization of their end-products by Reversed-Phase High-Performance Liquid Chromatography coupled to Electrospray Ionization Quadropole Time-of-Flight Mass Spectrometry (RP-HPLC-ESI-Q-ToF-MS). The workflow is outlined in Figure 1.

### 2.1. Photoprotection-Related Activities of Actinobacterial Isolates

Photodamage mechanisms include oxidative stress generation, which can be controlled with antioxidants [12] or prevented by reducing the adverse impact of UV radiation [10]. Since compounds with the ability to inhibit the radical chain reaction can serve as antioxidants, we evaluated the free radical scavenging capacity through two widely used methods (i.e., DPPH^•^ and ABTS^•+^) [34,35]. These methods comprise mixed mechanisms [36], since they provide an estimate of radical scavenging by involving hydrogen atom transfer (HAT) and single electron transfer (SET) mechanisms depending on the antioxidant nature, and they are differentially selective to the polarity of test compounds [35]. Regarding the blocking of UV radiation, given that both organic and inorganic UV filters act mainly by UV photons absorption [37], we also assessed the UV-absorbing capacity of the actinomycetes end-products.

#### 2.1.1. Radical Scavenging Capacity of Actinobacterial Isolates-Derived End-Products

**Figure 2:**
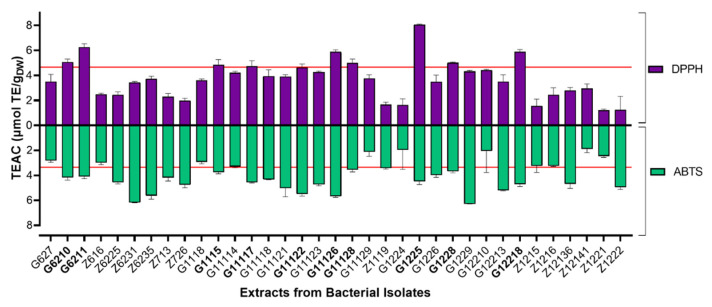
Antioxidant screening of actinobacterial methanolic extracts. Purple bars represent DPPH assays and green bars represent ABTS assays. Each TEAC (Trolox equivalent antioxidant capacity) value is expressed as the mean (n = 3), and error bars represent standard deviation (SD). Red lines indicate TEACs of 4.66 and 3.35 values for the DPPH and ABTS assays, respectively.

Most of the methanolic extracts (77.14%) behaved as radical quenchers against ABTS^•+^ above 50% radical scavenging, exhibiting an average TEAC value of 4.03 ± 1.18 μmol TE/g_DW_, whereas the radical scavenging for the DPPH^•^ assay was 3.59 ± 1.54 μmol TE/g_DW_ (Figure 2). The most promising isolates were selected considering those test extracts reaching scavenging capacity >50% in both assays (i.e., 4.66 μmol TE/g_DW_ for DPPH assays and 3.35 μmol TE/g_DW_ for ABTS assays), namely G6210, G6211, G1115, G11117, G11122, G11126, G11128, G1225, G1228, and G12218.

**Figure 3:**
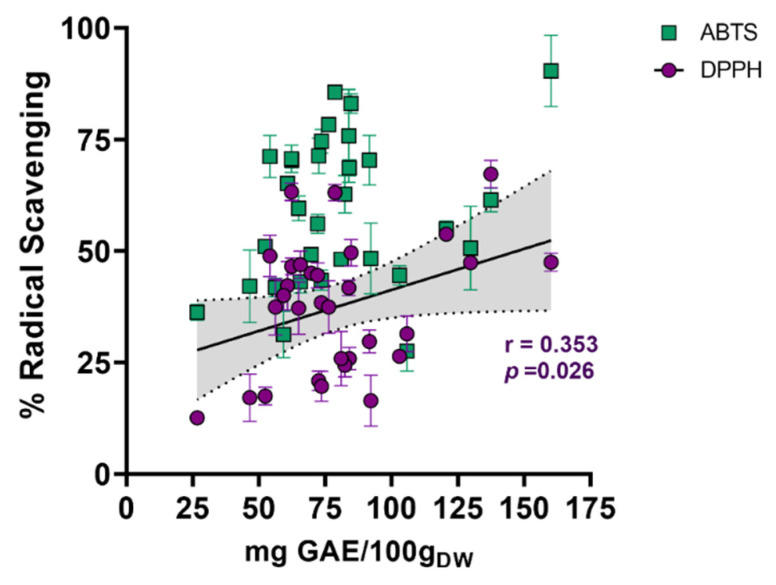
Correlation between antioxidant capacity assays and phenolic content. The results of ABTS and DPPH assays are shown as green squares and purple circles, respectively. *P*-value was determined by the Pearson correlation coefficient. Total phenolic content is expressed as milligrams of gallic acid equivalents per 100 g of dry weight (mg GAE/100g_DW_).

Since phenolics and flavonoids are compounds with well-recognized antioxidant capacity, we estimated their total content in all test methanolic extracts to examine their plausible participation in the measured radical scavenging ability. Although actinomycetes can synthesize flavonoids, the results showed that the tested methanolic extracts, unlike G11126 (TFC = 55.57 ± 10.97 mg QE/100g_DW_), did not show detectable levels of flavonoids. In contrast, the methanolic extracts contained a broad content of phenolics (i.e., from 26.69 to 160.15 mg GA/100 g_DW_), enabling a correlation analysis against the antioxidant results (Figure 3). Although the Pearson correlation coefficient was low for both assays (i.e., 0.353 and 0.250 for DPPH^•^ and ABTS^•+^, respectively), the correlation calculated for DPPH^•^ was statistically significant (*p* = 0.026; Figure 3).

#### 2.1.2. UV-Absorbing Capacity of Actinobacterial Isolates-Derived Extracts

**Figure 4:**
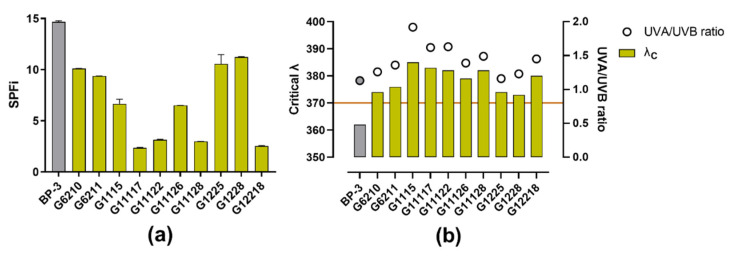
UV-absorbing profile of the actinobacterial methanolic extracts: (**a**) Results of SPFi calculations; (**b**) Results of the UV-A protection profile; the left *y*-axis shows the critical wavelength (λ_C_; the orange line indicates the threshold to claim broad-spectrum protection, i.e., 370 nm), and the right *y*-axis shows the UVA/UVB ratio. BP-3, which was used as a reference compound, is shown in gray.

The bacterial extracts with the higher antioxidant capacity were assessed for the UV-absorbing profile. Oxybenzone was employed as a reference UV filter. The bacterial extracts displayed diverse abilities to absorb UV radiation, ranging from 16.06% to 76.58% of the SPFi calculated for oxybenzone. Three isolates reached encouraging SPFis with values greater than 10 (in increasing order: G6210, G1225, and G1228; Figure 4a). Most of the available UV filters offer better UV-B protection; hence, compounds with UV-A protection demand more attention [38]. Interestingly, the actinobacterial extracts showed a λ_C_ higher than 370 nm, which is required to claim UV-A protection capability [33]. Additionally, the UVA/UVB ratio was the same or higher than the exhibited by oxybenzone (Figure 4b), achieving a 4-star rating according to the Boots Star Rating System [39]. These results indicate the occurrence of metabolites with a particular ability to absorb UV radiation between 320 and 400 nm over the UV-B spectrum.

### 2.2. In Vitro Safety Evaluation of the Photoprotective Actinobacterial Extracts

**Figure 5:**
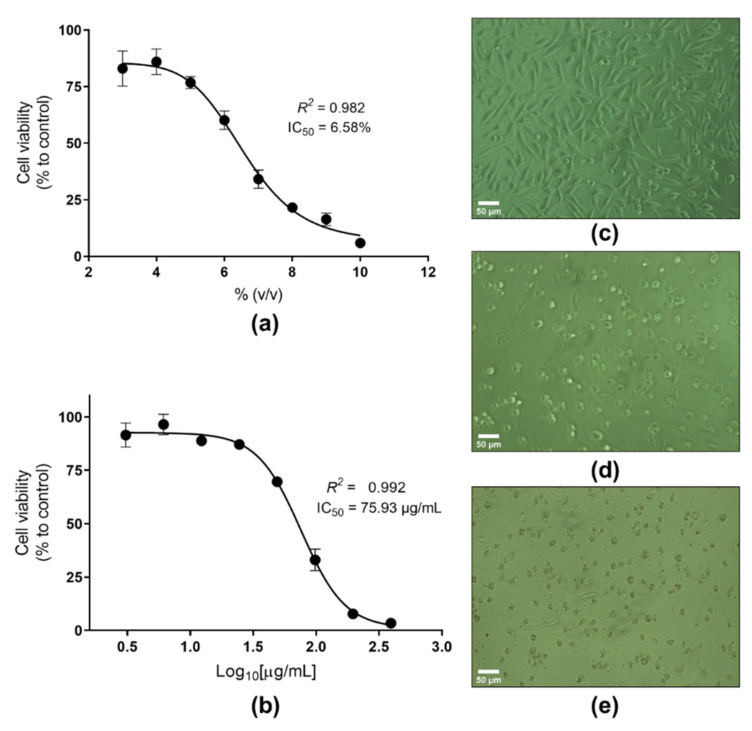
Cytotoxic evaluation of oxybenzone: (**a**) concentration–response curve of DMSO (between 10 and 3% *v*/*v*), (**b**) concentration–response curve of oxybenzone (between 400 and 3 µg/mL), (**c**) micrograph of HDFa cells in normal conditions, (**d**) micrograph of HDFa cells after 24 h the exposure to DMSO (9% *v*/*v*), (**e**) micrograph of HDFa cells exposed to oxybenzone (200 µg/mL) during 24 h.

Most commercially available UV filters have been associated with toxic effects, including oxybenzone [40]. We evaluated the cytotoxic effect of the selected photoprotective actinobacterial extracts (i.e., G6210, G6211, G1115, G11117, G11122, G11126, G11128, G1225, G1228, and G12218) on human dermal fibroblasts (HDFa cell line) and compared it to that shown by oxybenzone on the same cell line (Figure 5). Dimethyl sulfoxide (DMSO), at concentrations between 3 and 10% (*v*/*v*) (Figure 5a), was used as a cytotoxic reference substance. Oxybenzone displayed a cytotoxic effect with an IC_50_ of 75.93 µg/mL (Figure 5b). The cytotoxic effect was also observed at a morphological level, since HDFa cells exhibited a damaged cell morphology (i.e., loss of their spindly appearance, alteration in cell shape, membrane deformability; Figure 5e) compared with the usual visual appearance of these cells (i.e., crowded cells with elongated cell bodies and differentiated narrow ends; Figure 5c). A micrograph of HDFa after exposure to G1225 extract (i.e., the highest one antioxidant capacity) for 24 h is presented in Figure S1.

**Figure 6:**
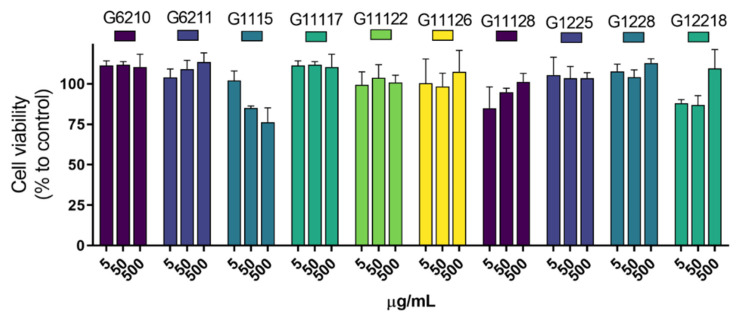
Cytotoxic evaluation of the actinobacterial crude extracts. The crude extracts were evaluated at 5, 50, and 500 µg/mL. Each value represents the mean (n = 3), and error bars represent standard deviation (SD). Cell viability was calculated with respect to untreated cells (control).

Regarding actinobacterial extracts, they were inactive (yielding far less than 50% cell viability reduction, Figure 6) and agree with drug discovery standards [41]. In addition, most of the extracts (90%) showed a cytotoxic effect higher than 12% on the HDFa cells. The G1115 extract exhibited the most cytotoxic action (reducing 23.84% of the cell viability at 500 μg/mL), whereas the cell viability was higher than 88% for the rest of the actinobacterial extracts.

### 2.3. Identification of Isolates with Photoprotective Potential by 16S rRNA Gene Sequencing

**Figure 7:**
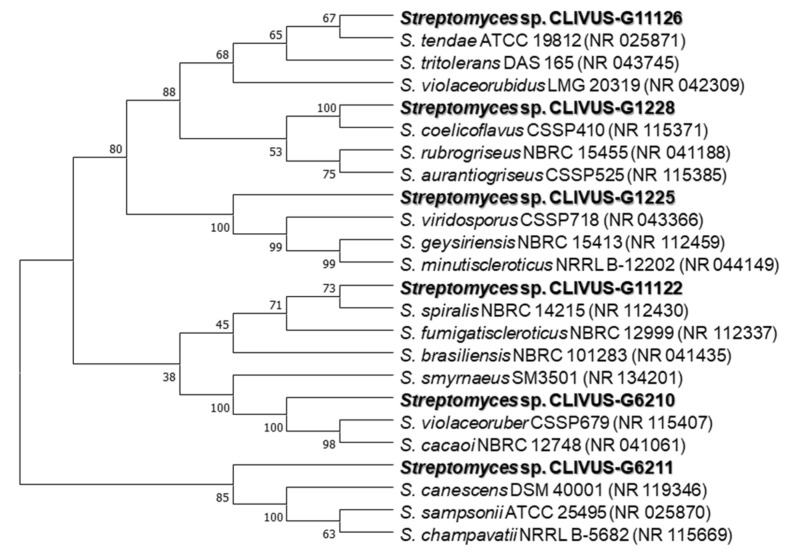
Phylogenetic tree of the bioactive streptomycetes isolates. The cladogram was built using the sequence of the 16S rDNA gene. The optimal tree is shown. The accession code of the blasted strains is shown in parentheses. The percentage of replicate trees with associated taxa clustered together in the bootstrap test (1000 replicates) is displayed next to the branches. All ambiguous positions were removed for each sequence pair (pairwise deletion option).

**Figure 8:**
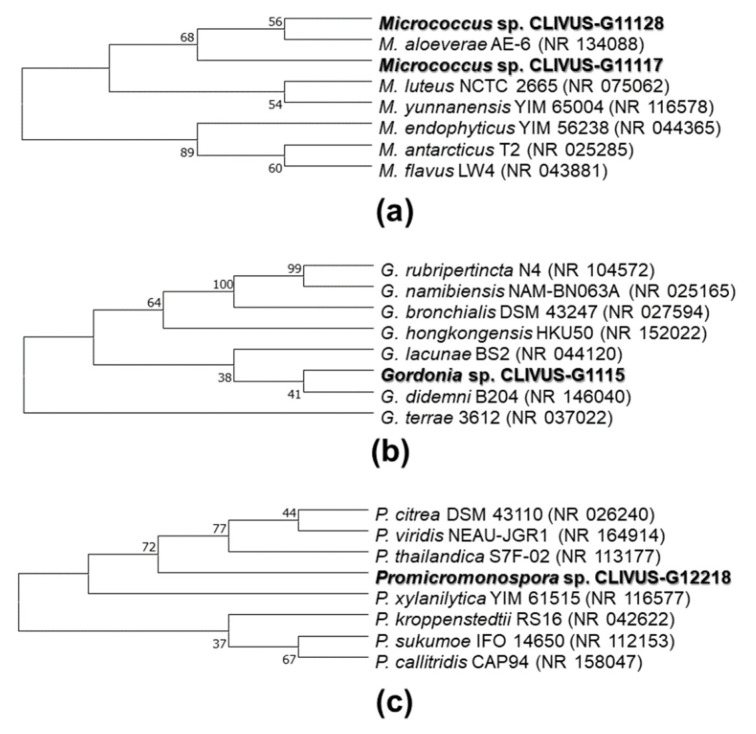
Phylogenetic tree of the bioactive non-streptomycete isolates: (**a**) cladogram for strains of genus *Micrococcus*, (**b**) cladogram for the strain of genus *Gordonia*, (**c**) cladogram for the strain of genus *Promicromonospora*. The cladograms were built using the sequence of the 16S rDNA gene. The optimal tree is shown. The accession code of the blasted strains is shown in parentheses. The percentage of replicate trees with associated taxa clustered together in the bootstrap test (1000 replicates) is displayed next to the branches. All ambiguous positions were removed for each sequence pair (pairwise deletion option).

The 16S rRNA gene was sequenced to identify those selected strains producing extracts with potential photoprotective activity. Then, a phylogenetic analysis with highly similar bacterial strains (chosen after a BLAST (Basic Local Alignment Search Tool) search) was conducted. The cladogram led to classifying the bioactive isolates among the actinobacterial genera *Streptomyces* (i.e., G11126, G1228, G1225, G11122, G6210, and G6211; Figure 7), *Micrococcus* (i.e., G11117 and G11128; Figure 8a), *Gordonia* (i.e., G1115; Figure 8b), and *Promicromonospora* (i.e., G12218; Figure 8c). The coding of the end-products and the names of the identified strains are presented in Table S1. To the best of our knowledge, this study is the first one reporting the isolation of these genera from *C. varians.*

**Figure 9:**
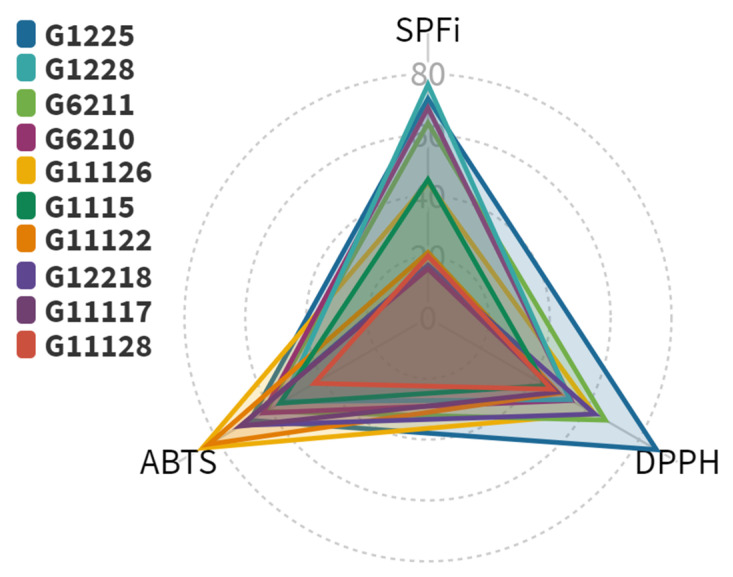
Radar chart to jointly illustrate the photoprotection-related activity results. The SPFi of each extract was normalized to the SPFi of BP-3, and the value is expressed as a percentage with respect to this reference UV filter. Data from DPPH and ABTS assays are expressed as radical scavenging capacity (RSC).

To consider the photoprotective potential shown by the actinomycetes end-products, we integrated the antioxidant and SPFi bioactivities into a radar chart (Figure 9). Hence, the photoprotective capability was ranked (in decreasing order) using the triangle area (Table S2) of each extract as follows: G1225, G1228, G6211, G6210, G11126, G1115, G11122, G12218, G11117, and G11128. Notably, the five most bioactive extracts came from *Streptomyces* strains, with *Streptomyces* sp. CLIVUS-G1225 as the top-ranked extract. The most bioactive isolate (i.e., CLIVUS-G1225) was related in a clade with *Streptomyces viridosporus, Streptomyces geysiriensis,* and *Streptomyces minutiscleroticus* (Figure 7). Recently, *S. geysiriensis* was considered as a later heterotypic synonym of *Streptomyces rochei* [42].

### 2.4. LC-MS-Based Characterization of Promising Microbial Extracts

**Figure 10:**
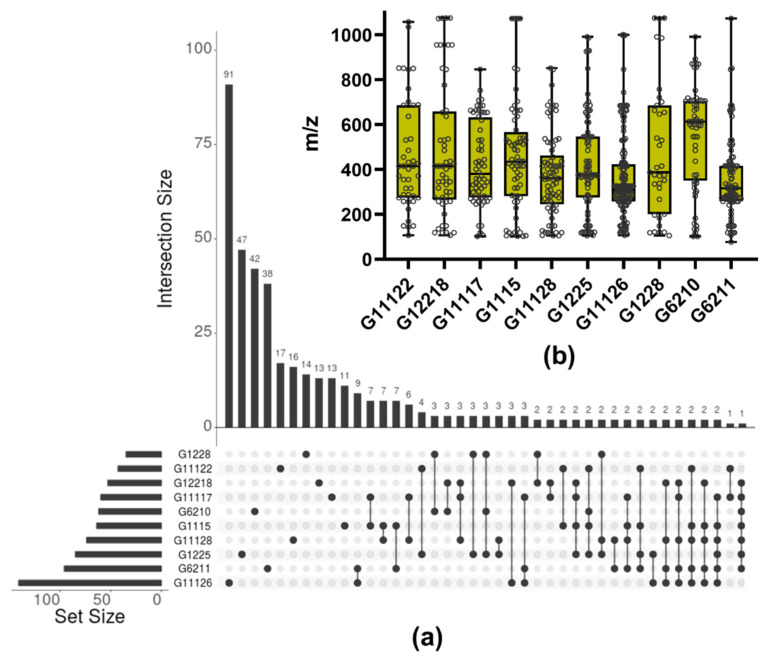
Overview of the LC-MS-based characterization of the bioactive actinobacterial extracts: (**a**) common and unique features are presented. The interconnected points indicate the extracts involved in the intersection; (**b**) *m*/*z* distribution of the features in each extract.

The selected actinobacterial extracts varied in terms of detected features content (i.e., from 35 for G1228 to 141 for G11126). To analyze the redundancy of the metabolite profile in the extract, considering that most of the selected extracts were from the same genus (i.e., *Streptomyces*), we inquire about the occurrence of the shared features in each extract (Figure 10a). Among *Streptomyces* strains, most of the features were unique, suggesting the presence of strain-specific metabolite occurrence. The biggest intersect was found to be between G6211 and G11126 (i.e., sharing nine features); however, G11126, in turn, presented the biggest number of strain-specific metabolites, which was followed by G1225.

Considering that molecular weight (MW) is a crucial physicochemical property associated with skin permeation [43,44], we explored the distribution of *m*/*z* features in each actinobacterial extract (Figure 10b). Compounds with low MW are associated with more significant skin absorption rates [44,45], while molecules with MW greater than >330 Da have shown a lower risk of skin absorption [46,47]. The extracts with the highest number of *m*/*z* ≥ 330 features were G6210 (80.65%) and G1225 (69.41%). Furthermore, based on the photoprotective capability shown by G1225 (Figure 8) and the number of detected features (Figure 9a), *Streptomyces* sp. CLIVUS-G1225 offers a distinct advantage and supports further analysis.

**Figure A1:**
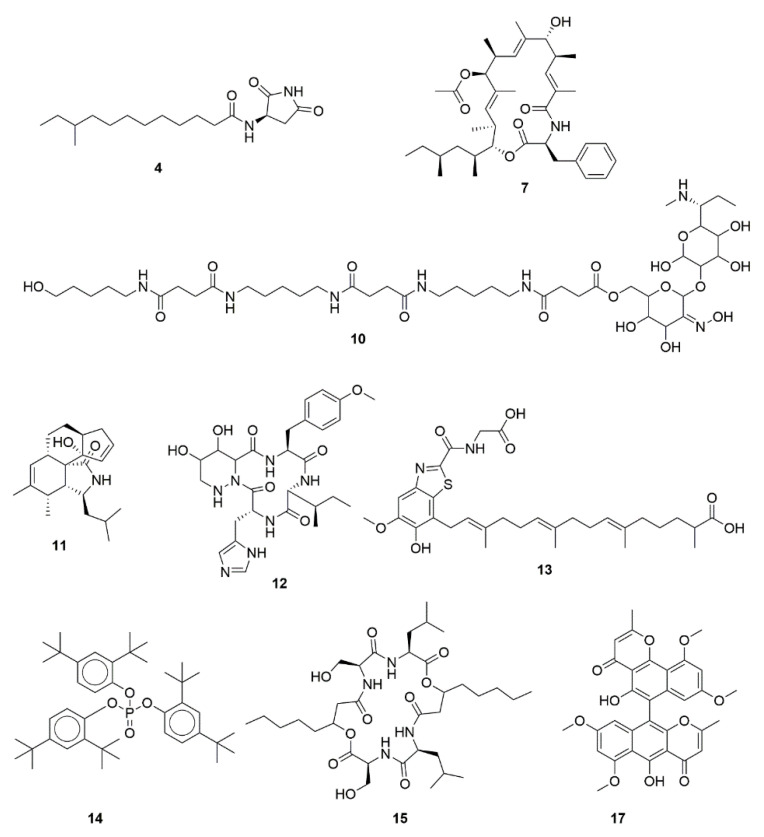
Chemical structures of the compounds identified at level 3 (**4**, **7**, **10**, **11**, **12**, **13**, **14**, and **15**) and level 4 (isonigerone **17**).

The resulting feature list from the MS-based annotation of the G1225 extract is summarized in Table 1. The features were identified at levels 3 (i.e., putative candidate) and 4 (i.e., unequivocal molecular formula), according to the confidence levels to communicate compound identity by high-resolution mass spectrometry (HRMS), as proposed by Schymanski et al. [48]. The masses ranged from 310.23 to 949.52 Da, which were commonly found among *Streptomyces*-derived metabolites (percentile 34th = 330.7 Da, Figure S2). At level 3, we annotated eight compounds (Table 2; Figure A1), from which two were isolated from *Streptomyces*, namely the sideromycin A (**10**) from *S. violaceus* DSM 8286 [49] and the glomecidin (**12**) from *S. lavendulae* H698 SY2 [50]. Although *tris*(2,4-di-*tert*-butylphenyl)phosphate (**14**) was annotated using StreptomeDB, it was isolated from a co-culture between the fungus *Bionectria* sp. and S. *lividans* [51]. In the same study, **14** was also isolated from a co-culture between *Bionectria* sp. and the bacterium *B. subtilis*, discarding the streptomycete as the source [51]. However, **14** has been previously identified in *Streptomyces* by LC-MS-based annotation [52]. In addition, we also putatively identified other compounds that were not previously reported in any streptomycete. Metabolites metacridamide A (**7**), periconiasin J (**11**), and icosalide B (**15**) were isolated from fungi species: *Metarhizium acridum* [53], *Periconia* sp. F-31 [54], and an unidentified fungus [55], respectively. Recent studies associated **15** with bacterial sources. It was firstly detected by MS-guide dereplication from the betaproteobacterium *Burkholderia gladioli* [56], and then, it was described that *Streptomyces* sp. JBS5-6 has the biosynthetic gene cluster of icosalide B [57]. For their part, bacillamidin C (**4**) and erythrazole A (**13**) were isolated from non-actinobacteria species, *Bacillus pumilus* RJA1515 [58] and *Erythrobacter* sp. SNB-035 [59], respectively.

Considering the importance of low skin absorption in skincare products, we inquired about the lipophilicity of the level 3 identified compounds by calculating the partition coefficient between *n*-octanol and water (cLogP; a well-established descriptor of a compound permeability [60]). In this sense, **10** and **12** stand out as putatively low-permeability compounds, which is a desired physicochemical characteristic focusing on the safety of skincare products [46,47].

**Figure 11:**
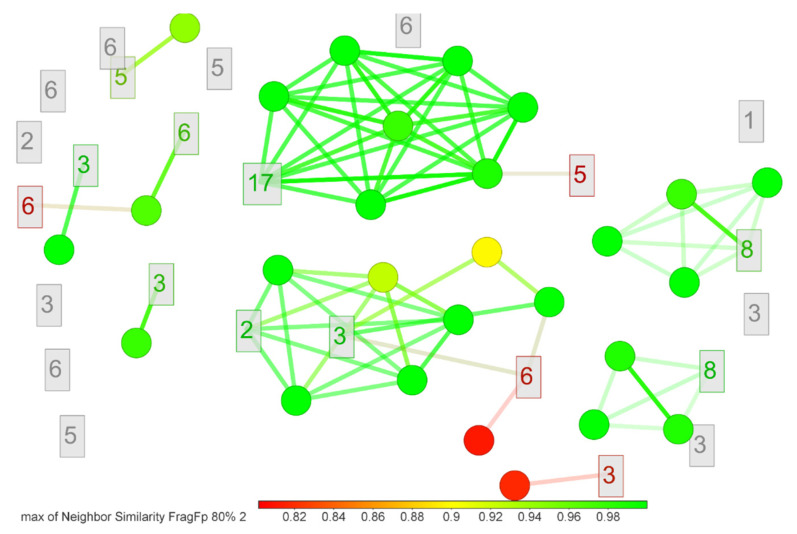
Isomers distribution among compounds identified at level 4 using the *FragFP* descriptor [62]. The similarity diagram was constructed using DataWarrior v.5.5.0 software.

Concerning the compounds identified at level 4 (i.e., **1**–**3**, **5**, **6**, **8**, **9**, **16**, and **17**), the number of isomers limited their further analysis (**51** options). However, to reduce the possibilities, closely related isomers were clustered to identify shared structural moieties representing each isomer (Figure 11). Hence, it was found that only for compound **17**, all isomers clustered into one group, with isonigerone as the representative compound. 

## 3. Discussion

The use of sunscreen and sunblock is an important action to avoid photodamage induced by overexposure to UV radiation. When developing an ideal photoprotective product, it is important to consider the role of oxidative stress in photodamage. At the same time, possible adverse effects on the cell populations to be protected must be avoided. This research aimed to find biological sources that produced compounds with in vitro photoprotection-related activities while remaining harmless against dermal fibroblasts. We found ten isolates from the associated microbiota of *C. varians* whose end-products exhibited in vitro free radical scavenging capacity, UV-absorbing ability, and non-toxicity against HDFa cells.

### 3.1. Actinomycetes as a Bioresource of Photoprotectants

One of the most critical bottlenecks in developing products from natural resources has been the “supply problem” [63]. Microorganisms, specifically symbionts, are a promising option to meet this challenge [64]. In this sense, one of the most relevant aspects of this study is the isolation and identification of microorganisms as a source of bioactive compounds. As mentioned above, sponges have been a vital resource providing bioactive compounds. In several cases, this success has been linked to their distinctive associated microbiota content. Therefore, the search for molecular entities with beneficial bioactivities from symbiotic microbiota has been growing recently. Although several *Streptomyces*-derived compounds showed photoprotection-related activities, we previously reported that very few studies focused on their application to prevent UV-induced skin damage. More interestingly, symbiotic *Streptomyces* derived from marine organisms have been the most poorly explored for their value regarding the production of photoprotectants. These considerations served as a relevant criterion for conducting the present study to disclose the potential of the associated microbiota of a sponge such as *C. varians*. In this context, as actinomycetes are perhaps the most important source of antibiotics [28] and, consequently, this bioactivity is found in most strains, our aim was oriented to expand such a bioactivity profile and identify isolates producing naturally occurring compounds with antioxidant and UV-absorbing capacities (i.e., photoprotection-related). Accordingly, we opted to screen extracts from bacterial isolates based on their ability to scavenge free radicals (i.e., ABTS^•+^ and DPPH^•^) and absorb UV radiation, as parameters to select bioactive isolates (Figure 1). Most of the selected isolates (six out of 10) were classified within genus *Streptomyces*, but these *Streptomyces* strains formed different clades (Figure 6). Considering that several studies have reported a wide chemodiversity in the *Streptomyces* genus [65], even within strains of the same species [66], a plausible estimation can be oriented to the fact that each strain could produce different metabolite profiles, involving relevant compounds with photoprotective potential.

According to the List of Prokaryotic names with Standing in Nomenclature (LPNS) [67], there are 675 validly published and correct names of *Streptomyces* species (plus 816 synonyms). From these numbers, 44 have been reported between 2018 and 2021 (date access 24 October 2021). This overview gives an idea of the vast biodiversity of the genus and clearly shows the importance of further exploration in the search for new streptomycetes. Except for strain CLIVUS-G1228 (forming a clade with *Streptomyces coelicoflavus* with a bootstrap value of 100%), the bootstrap values calculated for the remaining strains did not allow defining the respective *Streptomyces* species. These strains could even be new species; however, this fact will need to be further studied.

On the other hand, although *Streptomyces* continue offering valuable chances to find new bioactive compounds, rare actinomycetes (i.e., non-streptomycetes) are a distinct and relevant bioresource to search for highly valuable bioactive compounds [68]. Herein, four rare actinomycetes strains (i.e., *Micrococcus* sp. CLIVUS-G11128, *Micrococcus* sp. CLIVUS-G11117, *Gordonia* sp. CLIVUS-G1115, and *Promicromonospora* sp. CLIVUS-G12218) were also isolated. Compared to *Promicromonospora*, *Micrococcus,* and *Gordonia* microorganisms, species of genus *Streptomyces* are by far a well-studied natural source of bioactive metabolites. For instance, using the genus name as a keyword in the Scopus database (search performed on 12/Oct/2021), *Streptomyces* yields 43,097 hits, *Micrococcus* yields 18,105 hits, *Gordonia* yields 1,243 hits (without excluded *Gordonia* plant genus coincidences), and *Promicromonospora* yields 117 hits. This fact indicates that *Gordonia* and *Promicromonospora* genera are underexplored biological sources and gives an added research value to the strains we identify here (i.e., *Gordonia* sp. CLIVUS-G1115 and *Promicromonospora* sp. CLIVUS-G12218).

For the group of rare actinomycetes, compounds with antioxidant capacity, mainly pigments, have been reported in *Micrococcus* strains [69,70,71]. They have also been found to produce exopolysaccharides that can be used in various industrial sectors that require antioxidants [72,73]. A particularly interesting metabolite is 2,2′-[3-methoxy-1′-amyl-5′-methyl-4-(1″-pyrryl)]dipyrryl methene [69], which is a prodigiosin-like compound. This metabolite, besides being derived from *Streptomyces* [74], is known for its photoprotective properties [75]. Interestingly, a UV-specific repair enzyme has been isolated from *Micrococcus luteus*, which represents another promising alternative to using this bioresource to find photoprotection hits [76]. As expected, in the case of *Gordonia* and *Promicromonospra*, no studies investigated their photoprotective potential to our knowledge. However, carotenoids have been isolated from *Gordonia* [77], which are known for their antioxidative properties [78]. This information supports the idea that although understudied, these genera have photoprotective potential that justifies further study.

According to the bioactivities radar chart (Figure 9), the most promising strain was *Streptomyces* sp. CLIVUS-G1225. Although the strain types of the closest-related species were isolated from soil samples, some have also been isolated from marine sources: for instance, *S. rochei* from the marine sponge *Dysidea arenaria* [79] and *S. geysiriensis* from the marine sponge *Iotrochota* sp. [80]. Nevertheless, as far as we know, this is the first report of these *Streptomyces* strains isolated from the boring sponge *C. varians.* The taxonomic identity of *Streptomyces* sp. CLIVUS-G1225 needs to be further investigated using a whole-genome sequencing approach.

### 3.2. Scavenging, UV-Absorbing, and Cytotoxicity Potential of Actinomycetes End-Products

It has been shown that topical antioxidants can prevent and promote the restoration of UV-induced damage [81]. The purpose of an antioxidant is to stabilize a radical through HAT and/or SET mechanisms. Although the DPPH and ABTS assays are sensitive to both mechanisms (i.e., mixed-mode methods [82]), a solvent effect favors one over the other [83]. Hence, they allow the estimation of different types of radical scavenging compounds. Additionally, although both radicals have their radical at a hidden site, these steric hindrances are different. For example, the mechanism of reaction for DPPH with antioxidants is analogous to that of peroxyl radicals (ROO^•^; a type of UV-induced reactive oxygen species [84]) [82]. This fact explains the differences between the DPPH and ABTS results for the test extracts. In the case of the extracts of the selected isolates (i.e., G6210, G6211, G1115, G11117, G11122, G11126, G11128, G1225, G1228, and G12218), the results could be associated with the presence of compounds that are not limited by steric hindrance of either radical and might jointly exhibit radical scavenging by both HAT and SET mechanisms. We also found that the content of phenolics and flavonoids were not determinant in the antioxidant capacity of the test actinomycete-derived extracts. In fact, only one extract presented detectable levels of flavonoids, which agreed with previous studies [85,86,87].

Most UV filters approved for sunscreens absorb UV-B radiation thoroughly; very few are broad-spectrum (UV-B and UV-A), and even fewer are UV-A absorbers [88]. BP-3 is considered broad spectrum, although it protects more against UV-B and UV-AII (i.e., 320 to 340 nm) [89]. These features are consistent with the λ_C_ herein calculated (i.e., 362 nm, Figure 3b). As a result of its efficiency in absorbing UV radiation, it is one of the most widely used UV filters, not only in sunscreens [90], and consequently, it is considered a benchmark agent. However, a growing number of reports have raised concerns about the safety of BP-3 [90]. Our results were consistent with this fact, and BP-3 showed cytotoxic effects at the indicated concentrations. Indeed, the calculated IC_50_ of BP-3 corresponds to 47.46 µg/cm^2^ on cell monolayer in the in vitro assay, which is lower than the maximum allowed amount (i.e., 120–200 µg/cm^2^ [91]) in a recommended dose of sunscreen (i.e., 2000 µg/cm^2^, the quantity of sunscreen per unit of skin surface [92]).

Compared to BP-3, SPFi results of isolates G6210, G1225, and G1225 suggest the presence of compounds absorbing in the UV-B region (i.e., they reached 69.0, 79.1, and 76.3% of the SPFi calculated for BP-3). Furthermore, the λ_C_ values and the UVA/UVB ratio led to infer that they behave as broad-spectrum UV filters. Remarkably, these extracts were found as non-toxic against HDFa cells. UV-A injury is primarily produced in dermal tissue, where fibroblasts (a significant dermal cell population) are more susceptible to UV-A radiation than keratinocytes [93]. Therefore, selected actinobacterial extracts, with compounds absorbing preferably UV-A radiation and with an innocuous effect after exposure to fibroblast, constitute a starting point in the search for exploitable bioresources to obtain valuable photoprotectant agents. However, since our results are based on in vitro assays, further steps are required to validate these findings with in vivo methods.

### 3.3. Metabolite Profile of Promising Actinomycetes End-Products

We considered the hypothesis that most metabolites among *Streptomyces* strains would be shared. However, extracts contained mostly strain-specific metabolites, even among streptomycetes. This fact is consistent with the cladogram shown by *Streptomyces* strains (Figure 7), where each strain was placed in separate clades and with the well-known chemodiversity reported for the genus, including marine sponge-derived streptomycetes [94]. We decided to focus on the *Streptomyces* sp. CLIVUS-G1225 end-product, since it was found to be the most active one (Figure 9). Its metabolite profile presented a high number of features; most of these exhibited a MW higher than 330 Da (i.e., associated with low skin absorption). Skin absorption is an undesirable property in topically applied products, since it may result in interactions with unintended targets leading to side effects (e.g., endocrine disruptors [95]). These data located this *Streptomyces* strain in an advantageous position since the opportunity to find a biological producer of metabolites with preferred photoprotection-related properties, such as antioxidant, UV-absorbing, and safer characteristics (i.e., non-cytotoxic and high MW) could be favored.

Interestingly, several of the compounds annotated at level 3 in G1225 had not previously been reported in streptomycetes (i.e., **11**, **15**, **4**, and **13**). Moreover, **7, 11**, and **15** have been isolated from fungi. Even isonigerone (the compound that was found representative of the isomers associated with **17**; Figure A1), a naphthopyrone, was isolated from the marine-derived fungus *Aspergillus carbonarius* [96]. In *Streptomyces,* this type of naturally occurring compound has also been described [97]. There are other cases related to the biosynthesis of fungi-derived compounds by *Streptomyces*, such as aspergilazine A (isolated from the marine-derived fungus *Aspergillus taichungensis* [98] and *Streptomyces* sp. NRRL S-1868 [99]) and violapyrone J (isolated from the fungus *Cylindrocarpon* [100] and *Streptomyces somaliensis* SCSIO ZH66 [101]). Other intriguing examples that linked the biosynthetic pathways of fungi and *Streptomyces* are the β-lactams cephalosporin C (isolated from the fungus *Acremonium* [102]) and *O*-carbamoyl-deacetylcephalosporin C (isolated from *S. clavuligerus* [103]). Further examples involving cephalosporin-type compounds are reported by Higgens et al. [104]. These data could add further evidence to be further explored to the already recognized diversity in the biosynthetic machinery of streptomycetes and provide more relevance to the hypothesis of biosynthetic gene transfer from actinomycetes to fungi [105]. 

Regarding the bioactivity potential of the identified compounds, they have been poorly explored (Table 2). Compound **15** has shown in vivo anti-inflammatory effects [61]. Since inflammation is a pathophysiological response of UV insult [2], only **15** could be associated with photoprotection-related activities. Nevertheless, all the metabolites are aromatic and aliphatic amides, and several nitrogen-containing compounds have been associated with photoprotective capabilities, such as mycosporine-like amino acids [106] and betacyanins [107]. In fact, we have previously reported that amide-containing compounds are the most associated with the photoprotection-related activity, including antioxidant capacity, for *Streptomyces* [32]. For instance, thiazole-containing compounds derived from *Streptomyces*, such as **13**, have exhibited promising antioxidant capacity [108]. Therefore, we hypothesized that *Streptomyces* sp. CLIVUS-G1225 is a promising bioresource of underexplored compounds.

Microorganisms offer advantageous opportunities as a bioresource of bioactive compounds. Biotechnological tools could improve the target metabolite biosynthesis more easily compared to macroorganisms. In the case of *Streptomyces*, the implementation of approaches such as media engineering [109] and genetic engineering [109] for enhancing the production of value-added compounds have been successful. For instance, improvements of target metabolite production up to four-fold have been achieved in *Streptomyces* strains [110,111]. Ribosomal engineering is another interesting approach that has been proven to improve metabolite yield in *Streptomyces* [112]. Even though antibiotics are the main bioactivity found in *Streptomyces*-derived metabolites, several antioxidants have also been described [32], and only the undecylprodigiosinone has been characterized as a UV-absorbing compound from *Streptomyces* [113]. From these findings, *Streptomyces* is a valuable resource that can also be exploited to search for compounds with photoprotective properties leading to the development of novel sunscreens.

## 4. Materials and Methods

### 4.1. Chemicals and Reagents

The 2,2-diphenyl-1-picrylhydrazyl’(DPPH), 2,2′-azino-bis(3-ethylbenzothiazoline-6-sulfonic acid) (ABTS), L-ascorbic acid, gallic acid, Trolox, quercetin, Folin–Ciocalteu reagent (FCR), and methanol were purchased from MilliporeSigma (St. Louis, MO, USA). The 3-(4,5-Dimethylthiazol-2-yl)-2,5-Diphenyltetrazolium Bromide (MTT) was acquired from Thermo Fisher Scientific Inc. (Waltham, MA USA).

### 4.2. Sample Collection and Actinobacteria Isolation

*Cliona varians* specimens were collected by scuba diving from the Colombian Caribbean, Bahía de Taganga, Punta Venado (11°16′23.9″ N 74°12′24.9″ W) and Bahía de Santa Marta, Punta Betín (11°15′02.1″ N 74°13′16.0″ W), Magdalena, Colombia, at 13 m and 9 m depth, respectively. Around 10 g per sample (two individuals on each location) were placed in sterile plastic bags and transported on ice until processing. The taxonomic classification was performed by Dr. Sven Zea, confirming that all collected specimens were *C. varians*. The samples used in the present study have a Colombian origin, and they were obtained according to Amendment No. 5 to ARG Master Agreement No. 117 of 26 May 2015, granted by the Ministry of Environment and Sustainable Development, Colombia.

In the laboratory and aseptic conditions, the samples were quickly rinsed with abundant sterile seawater; then, the sponge tissue was homogenized using a mortar and pestle that were previously sterilized. Serial dilutions (1/10) up to 10^−6^ were prepared from the homogenate, and 100 μL of the dilutions 10^0^, 10^−3^, and 10^−6^ were plated, in duplicate, on Glucose Yeast Medium (GYM) and Zobell Marine Medium (Zobell) [114]. Media composition can be consulted in Table S3. Media were supplemented with nalidixic acid (50 μg/mL). Cycloheximide (100 μg/mL) was used to prevent fungal growth. The plates were incubated at 30 °C and carefully monitored for 8 weeks.

As actinobacteria-like colonies were picked, isolated, and individually grown to obtain pure cultures. Bacterial isolates were cryopreserved in their respective isolation medium with glycerol (30% *v*/*v*).

### 4.3. Submerged Fermentation and Crude Extracts Preparation

Based on 35 morphologically distinct actinomycetes isolates obtained from GYM (i.e., G627, G6210, G6211, G1118, G1115, G11114, G11117, G11118, G11121, G11122, G11123, G11126, G11128, G11129, G1224, G1225, G1226, G1228, G1229, G12210, G12213, and G12218) and Zobell (i.e., Z616, Z6225, Z6231, Z6235, Z713, Z726, Z1119, Z1215, Z1216, Z12136, Z12141, Z1221, and Z1222) medium, 10 mL of liquid submerged fermentation was done in the respective isolation media (i.e., GYM or Zobell). The seed culture was prepared inoculating a 55.81 mm^2^ plug from a 7-day-old lawn growth plate in 3 mL of broth. This broth culture was incubated at 30 ºC, 200 rpm agitation, for 7 days. From this seed culture, 1 mL was used to inoculate 9 mL of GYM or Zobell broth for 7 days at 30 ℃ and 200 rpm agitation). Then, each culture was lyophilized and submitted to an ultrasound-assisted extraction (frequency: 25 kHz; power effective: 200 W; temperature: <30 °C) with HPLC-grade methanol. Then, the extracted fraction was dried by a rotary evaporator and stored at 4 ℃ until the in vitro assays were completed. Each dry extract was resuspended in methanol according to the target tested concentrations.

### 4.4. Antioxidant Capacity Assays and Total Phenol and Flavonoid Content Measurements

The antioxidant capacity of the end-products was estimated using the 2,2′-azinobis-(3-ethylbenzothiazoline-6-sulfonic acid (ABTS) assay and the 2,2-di(4-tert-octylphenyl)-1-picrylhydrazyl (DPPH) assay, which are among the most used methods to estimate antioxidant capacity methods [34]. Assays were performed in 96-well plates in triplicate. Briefly, absorbance measurements were performed using an iMark^TM^ microplate reader (Bio-Rad Laboratories, Inc., Hercules, CA, USA), with Microplate Manager^®^ Software v6.3 (Bio-Rad Laboratories, Inc., Hercules, CA, USA).

#### 4.4.1. DPPH and ABTS Radical Scavenging Assays

The radical scavenging assays were performed as described previously [115] with some modifications. Briefly, a stock solution of DPPH at 0.2 mM in methanol and ABTS at 7 mM in distilled water were prepared. For the DPPH assay, 100 μL of microbial extracts were added to the 96-well plate; then, 100 μL of DPPH at 0.2 mM were suspended on each microbial extract (5 mg/mL, final concentration). The mixture was incubated for 30 min at room temperature and protected from light, and absorbance was measured at 515 nm.

For the ABTS assay, we follow Re et al. [116] with some modifications. Firstly, a mixture of ABTS with 2.45 mM potassium persulfate (final concentration) was allowed to stand under dark at room temperature for 16 h to generate ABTS radical cation (ABTS^•+^). The ABTS^•+^ solution was diluted with distilled water until an absorbance of 0.70 ± 0.02 at 735 nm. Then, 190 μL of the adjusted ABTS^•+^ solution was added to 10 μL of microbial extract (5 mg/mL, final concentration). Absorbance was recorded at 735 nm after 10 min of incubation at room temperature and protected from light.

The antioxidant capacity was expressed as radical scavenging capacity (RSC) and Trolox equivalents antioxidant capacity (TEAC). RSC was expressed as percentage (%) and calculated by Equation (1) as follows:

(1) $$\mathrm{Radical}\;\mathrm{scavenging}\;\mathrm{capacity}\;\left(\%\right)=\frac{A_{C}-A_{S}}{A_{C}}\times 100$$

where A_C_ is the absorbance of the blank (radical solution with solvent), and A_S_ is the absorbance of the radical solution mixed with the tested samples after incubation.

TEAC was expressed as μmol Trolox equivalents per gram of dry weight (μmol TE/g_DW_) and calculated by Equation (2) as follows:

(2) $$\mathrm{TEAC}\;\left(\mu \mathrm{mol}\;\mathrm{TE}/g_{\mathrm{DW}}\right)=\frac{T\;\left(\mu \mathrm{mol}/L\right)}{S\;\left(g/L\right)}$$

where T is the Trolox concentration obtained after interpolation using standard curves of Trolox from both assays (DPPH: *Y* = 2.167*X* − 0.5203; ABTS: *Y* = 3.066*X* − 1.356) and S is the concentration of the test sample.

#### 4.4.2. Total Phenol and Flavonoid Content

Total phenol content (TPC) was measured following the protocol reported by Magalhães et al. [117]. Briefly, 50 μL of the sample were suspended in each well, which was followed by 50 μL of the FCR (previously diluted 1:5 *v*/*v* in distilled water). Afterwards, 100 μL of NaOH at 0.35 M was added. Finally, the mixture was incubated at room temperature for 3 min, and the absorbance at 750 nm was recorded. The TPC was calculated using a gallic acid standard curve between 20.00 and 1.25 μg/mL. Data were expressed as mg GAE/100g_DW_.

The total flavonoid content (TFC) was measured as described by Buitrago et al. [115]. Briefly, 50 μL of ethanol was mixed with 10 μL of aluminum trichloride (10%) and 10 μL of sodium acetate (0.1 M). Once this mixture was dispersed in each well, we then added 70 μL of the samples. After 40 min under darkness at room temperature, the flavonoid content was estimated by measuring the absorbance at 415 nm. The TFC was calculated using a quercetin standard curve between 30.00 and 0.47 μg/mL. Data were expressed as mg QE/100g_DW._

#### 4.4.3. Determination of the In Vitro Sun Protection Factor and UV-Absorbing Profile

The absorbance spectra of the microbial extracts and BP-3 (1 mg/mL and 30 μg/mL, respectively, in quartz cuvettes) in the 290–400 nm region were recorded using a GENESYS™ 10S UV-Vis Spectrophotometer (Thermo Fisher Scientific Inc., Waltham, MA, USA). The in vitro Sun Protection Factor (SPFi) (Equation (3))was spectrophotometrically calculated according to Mansur et al. [118].

(3) $${\mathrm{SPF}}_{i}=CF\times \overset{320}{\underset{290}{\sum }}EE\left(\lambda \right)\times I\left(\lambda \right)\times A\left(\lambda \right)$$

where *CF* is the correction factor (i.e., 10), *EE* (*λ*) is the erythemal action spectrum, *I* (*λ*) is the solar intensity spectrum, and *A* (*λ*) is the absorbance value at wavelength *λ*. The values of *EE* (*λ*) × *I* (*λ*) are constants and were obtained from [119].

For the calculation of the Critical Wavelength (*λ_C_*), the following Equation (4) [39] was used:

(4) $$\int_{290}^{\lambda_{c}}A\left(\lambda \right)d\lambda =0.9\int_{290}^{400}A\left(\lambda \right)d\lambda$$

where *A* is absorption and *λ* wavelength.

The UVA ratio was calculated using the following Equation (5) [39]:

(5) $$\mathrm{UVA}/\mathrm{UVBratio}=\frac{\int_{320}^{400}A\left(\lambda \right)d\lambda }{\int_{290}^{320}A\left(\lambda \right)d\lambda }.$$

According to the UVA ratio result, the samples were classified using the start rating system [39].

#### 4.4.4. Sequencing of 16S rRNA Gene and Phylogenetic Analysis

Genomic DNA was extracted using a Quick-DNA Fungal/Bacterial Microprep kit (Zymo Research Corporation, Irvine, CA, USA) according to the manufacturer’s instructions. The 16S rRNA gene was amplified using the universal primers 27F and 1492R under the following PRC cycling conditions: initial denaturation at 94 °C for 3 min, followed by 30 cycles of 94 °C for 1 min, 50 °C for 1 min, and 72 °C for 2 min, with a final extension of 72 °C for 7 min. The amplification products were verified by agarose electrophoresis. The 16S rRNA gene sequences were blasted against the rRNA database with the megablast algorithm [120]. Consensus sequences were deposited in GenBank under the accession numbers OK598071 to OK598080. To find the closest neighbors used as reference sequences in the phylogenetic analysis, a Neighbor-Joining tree was conducted using MEGA version X [121] using the Tamura 3-parameter model. The reliability of the phylogenetic tree topology was assessed by the bootstrap test (1000 replications).

#### 4.4.5. Cytotoxicity Assay

The cytotoxic effect was evaluated on human primary dermal fibroblast (HDFa, ATCC^®^ PCS-201-012™, Primary Dermal Fibroblast; Normal, Human, Adult). HDFa were cultured in Dulbecco’s Modified Eagle Medium supplemented (DMEM) with 10% fetal bovine serum at 37 °C and 5% CO_2_. Cells were seeded into a 96-well plate at a density of 2 × 10^4^ cells/well and incubated overnight before exposure to treatment. The cells were treated 24 h with microbial extracts at 500, 50, and 5 μg/mL. Dimethyl sulfoxide (DMSO, between 1 and 10% *v*/*v*), methanol (0.5% *v*/*v*), and oxybenzone (between 400.00 and 3.13 μg/mL) were used as controls. After treatments, supernatants were replaced with fresh media with 3-(4,5-dimethylthiazol-2-yl)-2,5-diphenyltetrazolium bromide (MTT) at 0.5 mg/mL and incubated for 4 h [122]. Then, the MTT was removed, and DMSO was added to each well to dissolve formazan crystals. The amount of formazan was measured by its absorbance at 570 nm. Cell viability is calculated as the following formula (Equation (6)):

(6) $$\mathrm{Cell}\;\mathrm{viability}\;\left(\%\right)=\frac{A_{T}}{A_{U}}\times 100$$

where *A_T_* corresponds to the absorbance of treated cells and *A_U_* is the absorbance of the untreated cells.

#### 4.4.6. Data Analysis

Data are presented as mean ± standard deviation (SD). The antioxidant, cytotoxic, TPC, and TFC measurements were carried out in triplicate. For the inferential analysis, parametric statistics assumptions were verified; then, the differences were examined applying an ANOVA followed by a post hoc Tukey test at 95% confidence level. Finally, Pearson’s correlation coefficients were calculated to compare total contents and radical scavenging abilities.

#### 4.4.7. Metabolite Fingerprinting

The metabolite profiles of the extracts were recorded using an Agilent Technologies 1260 Liquid Chromatography system coupled to a Q-ToF 6545 time-of-flight quadrupole mass analyzer with dual Agilent jet stream electrospray ionization (AJS ESI) (Agilent, Santa Clara, CA, USA). The separation process consisted of an injection of 5 μL of the methanolic extracts onto a C18 column (InfinityLab Poroshell 120 EC-C18; 100 × 3.0 mm, 2.7 μm) (Agilent, Santa Clara, CA, USA) at 30 °C and a gradient elution composed of 0.1% (*v*/*v*) formic acid in Milli-Q water (Phase A) and 0.1% (*v*/*v*) formic acid in acetonitrile (Phase B) with a constant flow rate at 0.4 mL/min. The chromatographic method started at 15% of B for 3 min, which was followed by an increase up to 40% of B in 5 min; this concentration of B was held for 2 min, then, it was increased up to 70% B for 4 min with isocratic elution for additional 3 min, which was followed by a further increase up to 100% B over 4 min with isocratic elution for an additional 4 min. Finally, elution was fixed at 15% B in 4 min and held at 15% B for an additional 3 min. Mass spectrometric detection was performed in positive ion mode in a full scan from 70 to 1100 m/z. Two reference masses were used for mass correction throughout the analysis, i.e., m/z 121.0509 (C_5_H_4_N_4_) and m/z 922.0098 (C_18_H_18_O_6_N_3_P_3_F_24_). The AJS ESI source parameters comprised capillary voltage (3500 V), drying gas (8 L/min), gas temperature (325 °C), nebulizer pressure (50 psi), sheath gas temperature (350 °C), and sheath gas glow (11 L/min). The Q-ToF parameters involved fragmentor voltage (175 V), skimmer voltage (65 V), and octapole radiofrequency peak-to-peak voltage (OCT RF Vpp) (750 V). Detected features were annotated using StreptomeDB v3.0 [123] and Natural Products Atlas v2.0 [124] databases. To analyze the degree of structural relatedness between the isomers of the compounds identified at level 4 according to Schymanski et al. [48], we constructed a similarity diagram using Osiris DataWarrior v.5.5.0 (Idorsia Pharmaceuticals Ltd., Allschwil, Switzerland) software based on the *FragFp* descriptor [62].

## 5. Conclusions

This study is the first endeavor oriented to investigate the actinomycetes associated with the coral-reef boring sponge *C. varians* as a source of photoprotective compounds. *Streptomyces, Micrococcus, Gordonia,* and *Promicromonospora* were the genera with isolates showing promising results. *Streptomyces* sp. CLIVUS-G1225, *Streptomyces* sp. CLIVUS-G1228, *Streptomyces* sp. CLIVUS-G6211, *Streptomyces* sp. CLIVUS-G6210, and *Streptomyces* sp. CLIVUS-G11126 were found to be the most active in terms of photoprotection. Notably, these strains were not phylogenetically closely related and could not be defined at the species level, showing the need for further genetic analysis through whole-genome sequencing. *Streptomyces* sp. CLIVUS-G1225 exhibited higher performance in photoprotection-related activities at in vitro level, namely antioxidant (DPPH and ABTS radical scavenging capacity) and UV-absorbing, with the advantage of showing a non-cytotoxic effect. LC-MS-based characterization of its end-product revealed several compounds not previously reported from *Streptomyces* strains. Therefore, further work should concentrate on the isolation of these compounds, followed by their identification or structural elucidation, and assess their activity using in vivo methods for exploring their potential use in topical-applied products. Our findings provide the basis of another route to exploit the specialized metabolism of actinomycetes, especially concerning photoprotective compounds, postulating *Streptomyces* as a promising alternative bioresource to find substitutes to oxybenzone for sunscreens formulation.

## Figures and Tables

**Table 1:** Mass spectra data of the annotated features in *Streptomyces* sp. strain CLIVUS-G1225.

ID ^a^	RT ^b^	Molecular Formula	Adduct Type	*m*/*z*			Isomer Coincidence ^d^	Identification Level ^e^
				Experimental	Calculated	Δ (ppm) ^c^		
**1**	2.81	C_28_H_38_N_4_O_6_	[M+H]^+^	527.2838	527.2864	5.02	4	4
**2**	6.37	C_26_H_29_NO_5_	[M+2ACN+H] ^+^	518.2643	518.2650	1.39	7	4
**3**	14.50	C_22_H_26_O_6_	[M+H]^+^	387.1814	387.1802	2.96	13	4
**4**	15.22	C_17_H_30_N_2_O_3_	[M+2ACN+H] ^+^	393.2877	393.2860	4.22	1	3
**5**	15.69	C_26_H_28_O_6_	[M+H]^+^	437.1947	437.1959	2.66	5	4
**6**	16.30	C_24_H_30_O_6_	[M+H]^+^	415.2129	415.2115	3.35	9	4
**7**	20.00	C_37_H_55_NO_6_	[M+2ACN+H]^+^	692.4602	692.4633	4.47	1	3
**8**	20.12	C_32_H_54_N_4_O_7_	[M+ACN+H] ^+^	648.4338	648.4331	1.21	6	4
**9**	21.07	C_35_H_63_NO_3_	[M+H]^+^	546.4901	546.4881	3.69	3	4
**10**	21.79	C_42_H_75_N_7_O_17_	[M+ACN+H] ^+^	991.5576	991.5557	1.89	1	3
**11**	22.70	C_21_H_31_NO_2_	[M+2ACN+H] ^+^	412.2968	412.2959	2.22	1	3
**12**	22.85	C_27_H_37_N_7_O_7_	[M+2ACN+H] ^+^	654.3348	654.3358	1.48	1	3
**13**	22.93	C_31_H_42_N_2_O_7_S	[M+2ACN+H] ^+^	669.3346	669.3317	4.32	1	3
**14**	23.02	C_42_H_63_O_4_P	[M+H]^+^	663.4566	663.4537	4.38	1	3
**15**	23.34	C_34_H_60_N_4_O_10_	[M+H]^+^	685.4385	685.4382	0.48	1	3
**16**	23.44	C_22_H_38_O_2_	[M+ACN+H] ^+^	376.3202	376.321	2.19	3	4
**17**	25.29	C_32_H_26_O_10_	[M+ACN+H] ^+^	612.1849	612.1864	2.49	8	4

^a^ The annotated features were identified with consecutive Arabic numerals from **1** to **17** according to the elution order; ^b^ Retention time (min), ^c^ Mass measurement accuracy (ppm), ^d^ The number of isomers found in the databases; ^e^ Identification confidence levels according to Schymanski et al. [48]. Two-way profile (i.e., HPLC chromatogram × mass spectra) of the G1225 extract is presented in Figure S3.

**Table 2:** Bioactivity records of the main identified compounds at level 3 in *Streptomyces* sp. strain CLIVUS-G1225.

ID ^a^	Name	cLogP	Previously Described Bioactivities
**4**	Bacillamidin C	3.191	Antimicrobial, non-cytotoxic (evaluated against HepG2, A549, MDA-MB-231, SGC7901) [58].
**7**	Metacridamide A	8.213	Cytotoxic against Caco-2, MCF-7, HepG2/C3A [53].
**10**	Sideromycin A	−1.133	Antimicrobial [49].
**11**	Periconiasin J	3.132	Anti-HIV, non-cytotoxic (evaluated against MCF-7) [54].
**12**	Glomecidin	−2.695	Antifungal [50].
**13**	Erythrazole A	6.946	Non-cytotoxic (evaluated against non-small cell lung cancer cell lines) [59].
**14**	*Tris*(2,4-di-*tert*-butylphenyl)phosphate	13.777	Anti-inflammatory [61].
**15**	Icosalide B	2.551	Antiviral and moderate cytotoxic activities (evaluated against MDCK cells) [55].

^a^ Only eight compounds, namely **4**, **7**, **10**, **11**, **12**, **13**, **14**, and **15** could be putatively identified (i.e., level 3 according to Schymanski et al. [48]) in the G1225 extract.

## Data Availability

The data presented in this study are available in the article and Supplementary Materials.

## Supplementary Materials

The following are available online at https://www.mdpi.com/article/10.3390/md19120674/s1, Figure S1: Micrograph of HDFa cells exposed to G1225 (500 µg/mL) during 24 h, Figure S2: Distribution of *Streptomyces*-derived metabolite masses, Figure S3: Two-way profile (i.e., HPLC chromatogram × mass spectra) of the *Streptomyces* sp. CLIVUS-G1225 methanolic extract; Table S1: Identified actinomycetes strains, Table S2: Triangle area of the radar chart shown in the Figure 8, Table S3: Media composition.

## Appendix A

Chemical structures of the compounds identified at levels 3 and 4 (Figure A1).
