# Supplementary material for: Cliona varians-Derived Actinomycetes as Bioresources of Photoprotection-Related Bioactive End-Products
Source: Mar Drugs. 2021 Nov 27;19(12):674. doi: 10.3390/md19120674 (PMC8707384; doi:10.3390/md19120674)
Supplement: Supplementary file 1 [file marinedrugs-19-00674-s001.zip › Supplementary Materials.pdf]

Supplementary Materials

Cliona varians-derived actinomycetes as bioresources of photoprotective-related bioactive end-products

Jeysson Sánchez-Suárez [1, 2], Luisa Villamil [2], Ericsson Coy-Barrera [3] and Luis Díaz [1, 2, *]

[1] Doctorate in Biosciences, School of Engineering, Universidad de La Sabana, 250001 Chía, Cundinamarca, Colombia

[2] Bioprospecting Research Group, School of Engineering, Universidad de La Sabana, 250001 Chía, Cundinamarca, Colombia

[3] Bioorganic Chemistry Laboratory, Universidad Militar Nueva Granada, Cajicá 250247, Colombia

* Correspondence: luis.diaz1@unisabana.edu.co

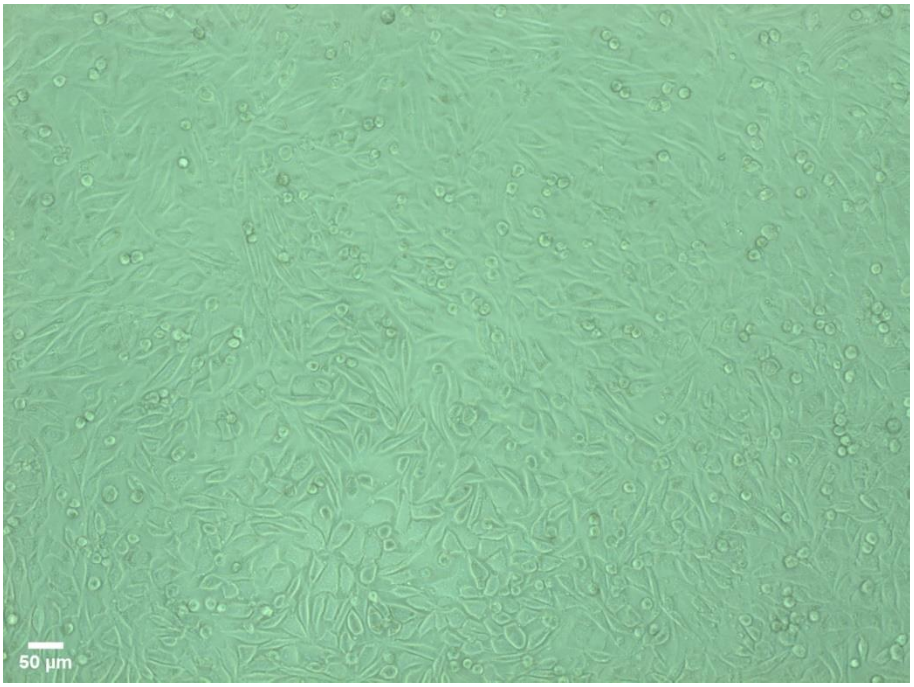

Figure S1. Micrograph of HDFa cells expose to G1225 (500 μg/mL) during 24 h.

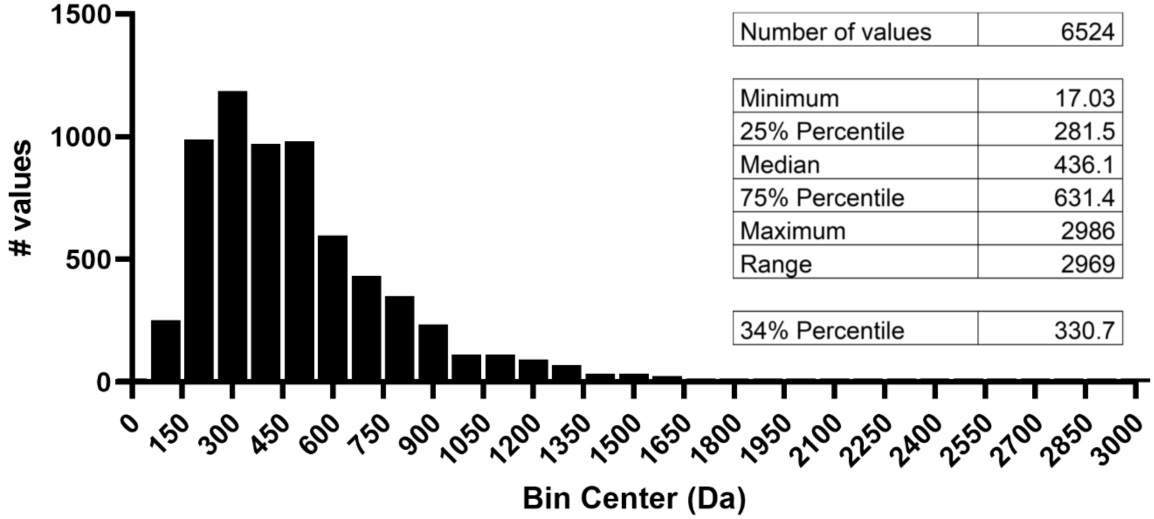

Figure S2. Distribution of Streptomyces-derived metabolite masses.

Table S1. Identified actinomycetes strains.

No.	End-products code	Strain name
1	G1225	*Streptomyces* sp. CLIVUS-G1225
2	G1228	*Streptomyces* sp. CLIVUS-G1228
3	G6211	*Streptomyces* sp. CLIVUS-G6211
4	G6210	*Streptomyces* sp. CLIVUS-G6210
5	G11126	*Streptomyces* sp. CLIVUS-G11126
6	G1115	*Gordonia* sp. CLIVUS-G1115
7	G11122	*Streptomyces* sp. CLIVUS-G11122
8	G12218	*Promicromonospora* sp. CLIVUS-G12218
9	G11117	*Micrococcus* sp. CLIVUS-G11117
10	G11128	*Micrococcus* sp. CLIVUS-G11128

Table S2. Triangle area of the radar chart shown in the Figure 8.

Actinomicyte strain	Triangle area
Streptomyces sp. CLIVUS-G1225	5542.19
Streptomyces sp. CLIVUS-G1228	4162.70
Streptomyces sp. CLIVUS-G6211	4112.90
Streptomyces sp. CLIVUS-G6210	4024.27
Streptomyces sp. CLIVUS-G11126	3302.97
Gordonia sp. CLIVUS-G1115	2280.98
Streptomyces sp. CLIVUS-G11122	1420.40
Promicromonospora sp. CLIVUS-G12218	1154.17
Micrococcus sp. CLIVUS-G11117	959.89
Micrococcus sp. CLIVUS-G11128	908.50

Table S3. Media composition.

Item	Glucose Yeast Media (GYM)	Zobell Marine Medium (Zoberll, ¼ strength)
D-Glucose	4	N/A
Yeast Extract	4	1.25
Malt Extract	10	N/A
NaCl	24	18
MgCl2	5.3	2
KCl	0.7	0.525
CaCl2	0.1	0.075
Agar	15	15
Peptone	N/A	3.75

The values are given in grams (g) per liter of medium.
